# Prevalence and Predictors of Silent Gastroesophageal Reflux Disease in Patients with Hypertension

**DOI:** 10.1155/2018/7242917

**Published:** 2018-04-23

**Authors:** He Suyu, Yijun Liu, Xu Jianyu, Guiquan Luo, Lipeng Cao, Xiaoqi Long

**Affiliations:** ^1^The Fourth Department of the Digestive Disease Center, Suining Central Hospital, Suining, Sichuan 629000, China; ^2^The Third Department of the Cardiology and Vascular Disease Center, Suining Central Hospital, Suining, Sichuan 629000, China; ^3^The Endoscopy Center, Suining Central Hospital, Suining, Sichuan 629000, China

## Abstract

**Background:**

Gastroesophageal reflux disease (GERD) without symptoms or silent GERD can be easily missed in patients with hypertension. We aimed to investigate the prevalence of GERD, specifically the prevalence of silent GERD in hypertensive patients, and to explore its possible predictors.

**Methods:**

Consecutive patients with hypertension referred to the cardiovascular clinic of Suining Central Hospital in 2016 were screened for this study. A Reflux Disease Questionnaire (RDQ) and an esophagogastroduodenoscopy (EGD) were employed for the evaluation of silent GERD. Included patients were divided into silent-GERD group and non-GERD control group. The demographic characteristics and antihypertensive agent prescriptions were collected and compared between the two groups.

**Results:**

The prevalence of silent GERD and GERD in patients with hypertension was 15.1% and 31.4%, respectively. 66 patients were included in the silent-GERD group, and 298 patients were included in the non-GERD control group. Abdominal obesity and untreated hypertension were positive predictors, while controlled hypertension was a negative predictor for silent GERD. The prescription of calcium channel blockers was not a predictor for it.

**Conclusions:**

High prevalence of GERD, specifically silent GERD, could be found in patients with hypertension. Abdominal obesity and untreated hypertension were positive predictors for silent GERD, while controlled hypertension was a negative predictor for it.

## 1. Introduction

Gastroesophageal reflux disease (GERD) is a common disorder defined as the presence of acid reflux-related symptoms or esophageal mucosal damage caused by the reflux of gastric contents into the esophagus [1]. Patients with GERD can be divided into three subgroups when esophagogastroduodenoscopy (EGD) is performed. GERD patients can be symptomatic without endoscopic lesions, symptomatic with endoscopic lesions, or asymptomatic with endoscopic lesions.

“Silent GERD” refers to esophageal mucosal injury (i.e., erosion, ulceration, or Barrett's esophagus) visible in EGD without typical or atypical GERD symptoms [2]. Researchers have discovered that a significant proportion of patients with GERD belong to this subgroup. According to the previous studies, approximately two-thirds of patients who had erosive esophagitis did not have reflux symptoms or more accurately these patients had “silent GERD” [3–7].

Hypertension is a cardiovascular disorder commonly observed in general practice [8]. Hypertension is a serious public health problem and is the most common cause for outpatient visits to physicians. According to the previous study, more than a quarter of the world's population is hypertensive, and this number is projected to increase to 29% by 2025 [9]. In China, hypertension has been identified as the second leading risk factor for total disease burden, immediately behind dietary factors [10].

It is common to observe silent GERD and hypertension concurrently, since many risk factors are shared by these two diseases. Risk factors including age, obesity, male sex, smoking, alcohol consumption, and education level have been reported to be significantly associated with both silent GERD and hypertension [11–17]. More importantly, previous studies have shown that silent GERD and hypertension not only coexist but they also mutually reinforce each other. On one hand, GERD can be a risk factor for hypertension. It has been reported that GERD is associated with an increased prevalence of hypertension relative to the general population [18]. On the other hand, hypertensive patients who take medications, such as calcium channel blockers, are at a higher risk of acquiring silent GERD [19].

Since both silent GERD and hypertension are common and share many common risk factors, we postulate that a higher prevalence of silent GERD exists in patients with hypertension. Because these patients have no symptoms, they cannot seek medical therapy for GERD. However, these patients are still at risk of developing GERD-related complications. Esophageal bleeding and stenosis may occur among patients with erosive esophagitis [5]. Most importantly, 25% of Barrett's esophagus cases and 40% of esophageal adenocarcinomas occur in patients without or with only minimal prior reflux symptoms [2, 20]. Therefore, these patients are an easily forgotten but clinically important population. The purpose of the present study was to investigate the prevalence of silent GERD in patients with hypertension and to identify the possible risk factors associated with the occurrence of silent GERD.

## 2. Materials and Methods

### 2.1. Study Population

Consecutive patients referred to the cardiovascular clinic of Suining Central Hospital between January 2016 and December 2016 were screened for this study. No patients enrolled in the study received remuneration. Requirements for participation included any one of the following: (1) diagnosis with primary hypertension (diagnosed before enrollment in the study and/or current use of antihypertensive medications); (2) classification in the New York Heart Association (NYHA) functional class I–III [21]; and (3) lack of heartburn, regurgitation, and/or other reflux-related symptoms (evaluated by questionnaire). The exclusion criteria were as follows: (1) secondary or identifiable hypertension; (2) first diagnosis with hypertension; (3) age less than 18 years or greater than 90 years and/or previous major psychotic episodes, mental retardation, dementia, severe visual or hearing abnormalities, or other illnesses that might render patients unable to complete the questionnaire or undergo the endoscopy (e.g., stroke); (4) history of other digestive disorders such as cirrhosis, chronic pancreatitis, Zollinger-Ellison syndrome, and malignancy; (5) history of upper gastrointestinal surgery; (6) abnormal upper gastrointestinal endoscopy findings for reasons other than reflux disease, such as esophageal varices and malignancy; (7) antacid therapy or treatment with antacid medications in the previous month; (8) refusal to participate; and (9) pregnancy.

### 2.2. Study Design

The present study was based on a standard protocol including the following three steps: (i) initial patient assessment, which included a complete collection of baseline characteristics and a face-to-face interview; (ii) the Reflux Diagnostic Questionnaire (RDQ); and (iii) endoscopy. All subjects needed to complete the self-reported RDQ questionnaire before endoscopy. Informed consent was obtained from all subjects before the protocol was administered.

#### 2.2.1. Initial Patient Assessment

*(1) Baseline Characteristics*. Baseline data were collected including gender, age, height, weight, body mass index (BMI), waist circumference, and educational status; routine laboratory parameters were assessed: blood and urine examination and blood biochemical analysis. The BMI was calculated as weight (kg)/height^2^ (m^2^). Weight status was categorized according to the Chinese criteria [22]: underweight (BMI < 18.5 kg/m^2^), normal (BMI ≥ 18.5 kg/m^2^ and BMI < 24 kg/m^2^), overweight (BMI ≥ 24 kg/m^2^ and BMI < 28 kg/m^2^), and obese (BMI ≥ 28 kg/m^2^). Waist circumference was divided into two categories according to the Chinese criteria [22]: normal (<85 cm for men and <80 cm for women) and central obesity (≥85 cm for men and ≥80 cm for women).

*(2) Face-to-Face Interview*. The interview obtained details on the following: smoking status, drinking status, history of diabetes mellitus, history of hyperlipidemia, family history of hypertension, and treatment with medications within the previous 4 weeks (antihypertensive medications, PPI, H_2_RAs, or other medications). The control status of hypertension was included when the subjects were receiving antihypertensive therapy. Regarding smoking, the participants were asked if they currently smoke. Smoking status was divided into current smokers and nonsmokers. For alcohol consumption, the amount of alcohol intake was recorded as the frequency of alcohol consumption over the past year, and alcohol consumption more than once a week was considered as “drinking alcohol.” Alcohol intake was categorized as drinking alcohol or not.

#### 2.2.2. Questionnaire

The Reflux Diagnostic Questionnaire (RDQ) is a reliable and well-validated instrument to diagnose GERD and can be easily applied by primary care physicians in a community setting [23]. The Chinese version of the RDQ has also been well validated and tested in a multicenter study including 10 hospitals in China [24]. The frequency and severity of heartburn, acid regurgitation, and dyspepsia were assessed using 12 items in the RDQ. The response was scored from 0 to 5 for each item. Each symptom was scored according to the frequency and severity (5-point scale). The highest score for one subject was 40. A diagnosis of silent GERD was made with an RDQ ≤ 12, while the diagnosis of symptomatic GERD was made with an RDQ > 12 [23, 24].

#### 2.2.3. Upper Gastrointestinal Endoscopy

Upper gastrointestinal endoscopy was employed to examine the presence of reflux endoscopic lesions. All upper gastrointestinal endoscopies were conducted after an overnight fast with standard endoscopes (XQ-260, Olympus Optical Co. Ltd., Tokyo, Japan) and by the same experienced endoscopist (Xiaojuan Jin). Diagnosis and classification of reflux esophagitis were based on the Los Angeles classification (grades A–D) [25]. Barrett's esophagus was diagnosed when columnar epithelium extended to the Z line, and intestinal metaplasia was confirmed histologically. The presence and extent of Barrett's epithelium were diagnosed based on the Prague C & M Criteria [26]. The shape of Barrett's epithelium was divided into three groups: island type, tongue type, and mixed type [27]. Barrett's esophagus with a circumferential length > 3 cm was defined as long-segment Barrett's esophagus, while that with a length of 1–3 cm was defined as short-segment Barrett's esophagus [28].

These criteria were consistently applied, and endoscopic pictures were reviewed by another experienced endoscopist (Xiaoqi Long). Generally, the final endoscopic diagnoses were made by the second endoscopist. A third endoscopist (Bin Yang) would join in and review all the endoscopic pictures if there is any discordance that occurred. The final endoscopic diagnoses will be made by a vote followed by the discussion among all these three endoscopists. Patients who returned for endoscopic reassessment for any reason were excluded from the analysis to prevent duplication of cases.

### 2.3. Diagnosis of Hypertension

Hypertension was defined as average systolic blood pressure ≥ 140 mmHg or diastolic blood pressure ≥ 90 mmHg or normal blood pressure in subjects currently taking antihypertensive medication [29].

#### 2.3.1. Measurement and Definitions

Measurement of blood pressure: two readings of systolic and diastolic blood pressure were taken from the right arm of each subject in a sitting position after a 10-minute rest using a standard clinical mercury manometer (Yuyue Medical Equipment & Supply Co. Ltd., Jiangsu, China). The mean value of the two readings was recorded as the blood pressure [30].

*(1) Definitions*. Antihypertensive treatment was defined as treatment with antihypertensive medication for greater than 20 days per month. Controlled hypertension was defined as normal blood pressure (systolic blood pressure < 140 mmHg and diastolic blood pressure < 90 mmHg) among subjects who were receiving treatment [29]. Untreated hypertension was defined as hypertension that had never been treated with a prescription medication or hypertension that had been treated, but the total treatment lasted less than 2 weeks.

### 2.4. Diagnosis of GERD and Silent GERD

GERD was diagnosed based on the presence of reflux symptoms and/or the presence of reflux esophagitis or Barrett's esophagus. Silent GERD was defined as the presence of reflux endoscopic lesions (erosive esophagitis or Barrett's esophagus) but with an RDQ score ≤ 12. A subject with asymptomatic GERD was defined according to the RDQ score (>12) [24]. Patients who were classified as having GERD according to the questionnaire (RDQ > 12) but did not have evidence of reflux lesions were diagnosed as having GERD.

### 2.5. Ethical Considerations

The study was performed in accordance with the principles of the 1975 Declaration of Helsinki and approved by the Ethical Committee of Suining Central Hospital. Informed consent was obtained from each participant.

### 2.6. Statistical Analysis

All analyses were performed using SPSS software (version 17.0; SPSS, Chicago, IL, USA). Categorical data were presented as percentages, while continuous data were presented as means with standard deviations. Chi-square tests and independent sample *t*-tests were used to analyze categorical and continuous variables. Logistic regression methods were applied for variables for the multivariate analysis. The odds ratios (OR) and 95% confidence intervals (CIs) were obtained. Two-tailed *P* value < 0.05 was considered to be statistically significant.

## 3. Results

### 3.1. Prevalence of Silent GERD and GERD in Patients with Hypertension

**Figure 1:**
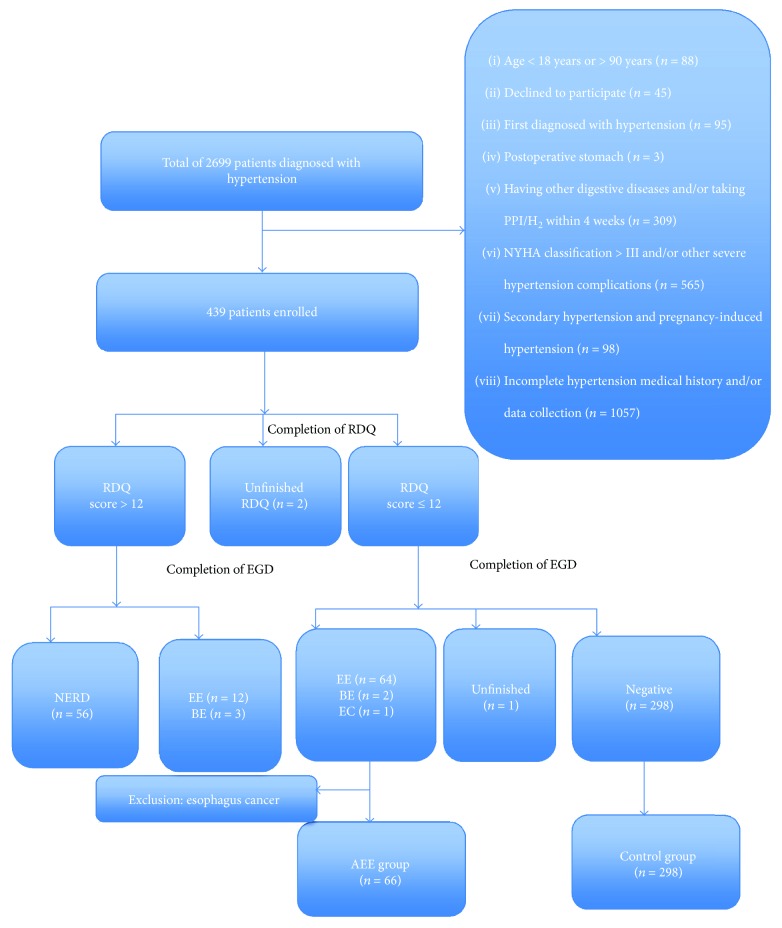
Flow chart of patients enrolled into this study. RDQ: Reflux Disease Questionnaire; EGD: esophagogastroduodenoscopy; BE: Barrett's esophagus; GERD: gastroesophageal reflux disease; EE: erosive esophagitis; NERD: nonerosive reflux disease.

Of 2699 patients with hypertension for this study, a total of 439 patients met the inclusion criteria. Among these patients, 2 did not complete the questionnaire effectively. One patient did not complete the EGD examination. Based on the results of the RDQ test and the EGD, the prevalence of silent GERD was 15.1% (66/436). Among these patients, 87.9% (58/66) were Los Angeles (LA) grade A, 9.1% (6/66) were LA grade B, and 3.0% (2/66) were Barrett's esophagus (Table 1). Both of these two subjects with Barrett's esophagus were island type and short segment of Barrett's esophagus. Symptomatic GERD was diagnosed in 16.3% (71/436) of patients. Therefore, the final prevalence of GERD in patients with hypertension was 31.4% (137/436) in our study (Figure 1).

### 3.2. Comparison of Demographic and Other General Factors between Silent-GERD and Non-GERD Control Groups

There were 66 subjects in the silent-GERD group and 298 subjects in the non-GERD control group. The demographics, lifestyle factors, family histories of hypertension, classifications and complications of hypertension, and control rates of hypertension were compared in Table 2. There were no differences between the two groups regarding mean age, gender, smoking status, alcohol drinking status, BMI, family history of hypertension, or classifications and complications of hypertension. Both the proportions of patients with higher education levels (≥12 years) (12.1% versus 23.8%) and those with controlled hypertension (15.8% versus 26.2%) were significantly lower in the silent-GERD group compared to the non-GERD control group (*P* = 0.037 and *P* = 0.012, resp.). Conversely, the waist circumference but not the BMI was significantly larger in the silent-GERD group compared to the non-GERD control group (87.1 ± 10.8 cm versus 85.1 ± 9.6 cm, *P* = 0.034) (Table 2).

Regarding the comparison of the antihypertensive agents and other common comedication prescriptions, no differences were observed between the two groups, except for the number of prescriptions of calcium channel blockers and the proportion of subjects taking no antihypertensive agents. Both the proportions of patients taking prescriptions of calcium channel blockers (66.7% versus 52.3%) and those taking no antihypertensive agents (9.1% versus 2.7%) were significantly higher in the silent-GERD group compared to the non-GERD control group (*P* = 0.034 and *P* = 0.014, resp.) (Table 3).

### 3.3. Comparison of Factors between the Silent-GERD Group and the Non-GERD Control Group Using Logistic Regression Analysis

Multivariate analysis was performed for the following parameters: education level, abdominal obesity, treatment with prescriptions of calcium channel blockers, controlled hypertension, and untreated hypertension. Statistically significant differences were identified in the univariate analysis (results in Section 3.2).

The assignments of the demographic data and silent-GERD-related factors in hypertensive patients are shown in Table 4.

Three significant independent risk factors for the occurrence of silent GERD were identified after adjusting for other factors. Abdominal obesity (OR = 11.35, 95% CI: 4.92–26.18, *P* ≤ 0.001) and untreated hypertension (OR = 17.50, 95% CI: 3.65–83.87, *P* ≤ 0.001) were positive predictive factors for the occurrence of silent GERD, while controlled hypertension was a negative predictive factor for silent GERD (OR = 0.02, 95% CI: 0.01–0.09, *P* ≤ 0.001) compared with the control group (Table 5).

## 4. Discussion

The primary findings of this study are that patients with hypertension have a high prevalence of GERD, specifically silent GERD. Three risk factors including abdominal obesity, controlled hypertension, and untreated hypertension are associated with the occurrence of silent GERD in patients with hypertension.

The high prevalence of silent GERD in patients with hypertension deserves more attention. How can we screen the right patients to do the right examinations and therapies? In this study, multivariate analysis showed that three factors were predictive for silent GERD compared with the control group. To date, no study has been performed to investigate the possible risk factors for the occurrence of silent GERD in patients with hypertension. Only studies which were done in subjects attending health examinations or in subjects diagnosed with GERD could be found. A recent study that was conducted in subjects attending health examinations in Taiwan reported that male sex and hiatus hernias were positive risk factors for silent GERD, while the active infection of *H. pylori* was negatively associated with the occurrence of silent GERD [7]. Male sex, a BMI over 25, smoking, and/or drinking were also reported to be positive or negative factors for the occurrence of asymptomatic esophagitis in other studies done in Korea [11].

These results differ slightly from our results. Different study populations might be the primary reason for the difference since our target population was patients with hypertension instead of patients undergoing health examinations. It is well known that older age, male sex, and smoking are also risk factors for hypertension, which may explain why we did not find a significant difference related to these factors between the silent GERD group and the control group. In addition, different diagnostic criteria for silent GERD may also contribute to the differing result discrepancy. In previous studies, silent GERD was defined as RDQ scores < 5 or <3 [7, 14]. This means that some subjects whose RDQ scores were ≥5 or ≥3 but ≤12 were excluded since they were neither totally asymptomatic nor qualified to be diagnosed with symptomatic GERD. In our study, RDQ scores ≤ 12 were employed as the diagnostic criteria for silent GERD since patients with minor symptoms or atypical symptoms were rarely diagnosed with GERD, let alone seek medical help for it. This means the clinical result for patients belonging to this group could be the same as patients without any symptoms related to GERD. Therefore, it is more reasonable to include them in the silent-GERD group. We did not investigate the status of *H. pylori* in silent GERD, since its role in GERD is still controversial.

Our study also showed that abdominal obesity but not BMI was positively associated with the occurrence of silent GERD. Dozens of previous studies have demonstrated a positive association between increased BMI and the presence of erosive esophagitis [31]. We postulate that silent GERD, as a subgroup of GERD, shares some common characteristics and pathogeneses with GERD. However, it may also have its own unique characteristics and pathogenesis. This is consistent with the previous studies. Robertson et al. reported that shorter LES was found in asymptomatic volunteers with large waist circumferences compared to those asymptomatic volunteers with small waist circumferences [32]. It is known that waist circumference is the main indicator for abdominal obesity. More importantly, elevated intra-abdominal pressure from abdominal obesity may produce mechanical distortions of the gastroesophageal junction [33]. Another study analyzing the clinical characteristics of asymptomatic GERD also reported that abdominal obesity tended to be associated with silent GERD [7]. Therefore, the impact of abdominal obesity on the occurrence of silent GERD might be much more important than that of overall obesity.

It is also worth noting that the prescription of calcium channel blockers in this study was significantly higher in the silent-GERD group (*P* = 0.034) compared to the non-GERD control group. This finding is consistent with an early 6-year follow-up study in Japan which found that the prescription of calcium channel blockers in patients with newly developed GERD is significantly higher than that in patients who did not develop GERD [34]. More recently, another study done in India also showed that the prescription of calcium channel blockers is positively associated with the occurrence of GERD [35]. Although our study also found a higher number of prescriptions of calcium channel blockers in patients with silent GERD compared to the control, it was not a risk factor for the occurrence of silent GERD after adjusting for other factors. However, our sample size is too small. Further studies are still required to confirm the association between calcium channel blockers and silent GERD, since calcium channel blockers are the most commonly prescribed antihypertensive agents in patients with hypertension.

Another interesting phenomenon that we have found in our study is that the prevalence of symptomatic GERD is also higher in patients with hypertension. Symptomatic GERD was diagnosed in 16.3% (71/436) of patients in our study which was higher than symptomatic GERD in normal subjects without hypertension. According to the previous study, the prevalence of symptomatic GERD was reported to be 5.6% in subjects without hypertension [3]. Although patients with symptomatic GERD are not the focus of this study, this higher prevalence deserves some attention. We postulate that the actual prevalence of symptomatic GERD might be higher since we have excluded subjects who took antacid agents in the previous month in this study. We will investigate it further in our next study.

Our study has some notable features. First, we included both patients with erosive esophagitis and patients with Barrett's esophagus in our silent-GERD group. Although patients with Barrett's esophagus but without symptoms should be included in the silent-GERD group as defined by Fass and Dickman [2], most relevant studies did not include patients with Barrett's esophagus [7, 11, 12]. It is true that the prevalence of Barrett's esophagus is only approximately 1% in China [36]. However, it is well known that Barrett's esophagus is associated with the occurrence of esophageal adenocarcinoma. More importantly, up to 40% of patients with esophageal adenocarcinoma can be asymptomatic [20]. Second, RDQ scores < 12 were set as one of the diagnostic criteria for silent GERD for the first time, since patients with minor symptoms who do not qualify for the diagnosis of GERD may have the same clinical results as the patients without any symptoms. Furthermore, a score of 12 is universally accepted as a diagnostic criterion for GERD in studies with Chinese subjects [3, 24, 37, 38]. However, there are also several potential limitations of this study. First, we did not include all subjects with hypertension. The strict inclusion criteria possibly resulted in some bias. As we know, there were some patients with severe complications of hypertension, such as patients with NYHA class IV. It would be risky for these patients to undergo invasive examinations. Additionally, we did not include subjects diagnosed with hypertension for the first time, since it is not feasible to evaluate the possible influence of antihypertensive agents in these patients. Second, the sample size is small. We screened only 66 patients with silent GERD. However, all of the patients presented here were well defined and homogeneous. Third, we did not compare the patients with both positive endoscopic findings and symptoms with those patients with positive endoscopic findings but no symptoms. After screening with RDQ and endoscopy, only 15 subjects were symptomatic and had positive endoscopic findings. The sample size is too small to compare with the patients in the silent-GERD group. However, it would be meaningful to compare these two groups. We plan to investigate this in our next study. Fourth, regarding the smoking status and alcohol consumption, we did qualitative analysis but not quantitative analysis, although both smoking and alcohol consumption have been proved to be possible risk factors for GERD in previous studies [39, 40]. Whereas the relationship between smoking, alcohol consumption, and silent GERD is still uncertain or more accurately still controversial, some studies even reported recent alcohol consumption may reduce the risk of erosive esophagitis and Barrett's esophagus [41]. We plan to further investigate the relationship between these lifestyle factors and silent GERD in our next study. Besides, we did not investigate the clinical prognosis of patients with silent GERD. It was not the focus of this study. However, it is important, and a long follow-up period is required. It should be investigated in our future study.

In conclusion, we have shown that patients with hypertension have a high prevalence of GERD, specifically silent GERD. Abdominal obesity and untreated hypertension were positively associated with silent GERD, while controlled hypertension was negatively associated with it.

## Figures and Tables

**Table 1:** Endoscopic findings of the subjects in the silent GERD group.

Endoscopic findings	Total number (*n*)	% (*n*/66)
GERD	66	
LA grade A	58	87.9%
LA grade B	6	9.1%
LA grade C	0	0
LA grade D	0	0
Barrett's esophagus	2	3.0%
Esophageal cancer	1	

LA: Los Angeles.

**Table 2:** Baseline characteristics of the silent-GERD group and non-GERD controls.

Characteristics	Silent GERD (*n* = 66)	Controls (*n* = 298)	*F*/*X*^2^ value	*P* value
*Age (mean ± SD), years*	64.7 ± 11.2	63.9 ± 10.1	0.184	0.669
*Male, n (%)*	36 (54.5)	151 (50.7)	0.325	0.569
*Education level*				
≥12, years	8 (12.1)	71 (23.8)	4.356	0.037^❖^
<12, years	58 (87.9)	227 (76.2)		
*Smoking, n (%)*	20 (30.3)	81 (27.2)	0.263	0.608
*Alcohol drinking, n (%)*	11 (16.7)	44 (14.8)	0.152	0.696
*Height (mean ± SD), cm*	164.6 ± 8.4	164.0 ± 8.7	1.369	0.243
*Weight (mean ± SD), kg*	68.1 ± 11.7	166.8 ± 12.0	0.663	0.416
*BMI (mean ± SD), kg/m^2^*	24.8 ± 3.4	24.2 ± 3.6	0.572	0.450
*Waist circumference (mean ± SD), cm*	87.1 ± 10.8	85.1 ± 9.6	4.398	0.034^❖^
*Family history of hypertension*	19 (28.8)	91 (30.5)	0.078	0.779
*Classification of hypertension*				
Grade 1, *n* (%)	9 (14)	47 (16)	0.189	0.664
Grade 2, *n* (%)	20 (30)	90 (30)	0.000	0.987
Grade 3, *n* (%)	21 (32)	83 (28)	0.416	0.519
Others, *n* (%)	16 (24)	78 (26)	0.105	0.746
*Complications of hypertension*				
Dyslipidemia, *n* (%)	28 (42.4)	121 (40.6)	0.074	0.786
DM, *n* (%)	18 (27.3)	57 (19.1)	2.191	0.139
CAD, *n* (%)	15 (22.7)	77 (25.8)	0.277	0.599
Stroke, *n* (%)	5 (7.6)	24 (8.1)	0.017	0.897
Chronic renal dysfunction, *n* (%)	3 (4.5)	11 (3.7)	0.000	1.000
*Controlled hypertension, n (%)*	9 (15.8)	78 (26.2)	6.285	0.012^❖^

BMI: body mass index; DM: diabetes mellitus; CAD: coronary artery disease. *P* < 0.05 was selected as a significant level; ^❖^*P* < 0.05 compared with the control group.

**Table 3:** Antihypertensive agents and other common comedication prescriptions of the two groups.

Drugs	Silent GERD (*n* = 66)	Non-GERD controls (*n* = 298)	*F*/*X*^2^ value	*P* value
*Antihypertensive agents*				
Calcium channel blockers	44 (66.7)	156 (52.3)	0.474	0.034^❖^
ARBs	20 (30.3)	95 (31.9)	0.062	0.803
ACEIs	13 (19.7)	62 (20.8)	0.041	0.840
*β*-blockers	15 (22.7)	70 (23.5)	0.018	0.895
Diuretics	6 (9.1)	28 (9.4)	0.004	0.949
ARB/ACEI and diuretic compounds	3 (4.5)	10 (3.4)	0.011	0.917
Other antihypertensive agents	2 (3.0)	11 (3.7)	0.000	1.000
No antihypertensive agents	6 (9.1)	8 (2.7)	5.996	0.014^❖^
*Comedication*				
Lipid-lowering agents	22 (33.3)	108 (36.2)	0.199	0.655
Antiplatelet agents	14 (21.2)	78 (26.1)	0.705	0.401
Antidiabetic medications^♦^	11 (61.1)	32 (56.1)	0.138	0.710

All results expressed as “*n* (%).” ARB: angiotensin receptor blockers; ACEI: angiotensin-converting enzyme inhibitors. ^❖^*P* < 0.05 compared with the control group. ^♦^Based on 75 patients with diabetes mellitus.

**Table 4:** Assignment table of variables in patients with hypertension.

Factors	Variables	Explanation of assignments
Higher education level	X1	<12 = 0, ≥12 = 1
Abdominal obesity	X2	No = 0, yes = 1
Calcium channel blockers	X3	Without prescription = 0, prescription = 1
Controlled hypertension	X4	Uncontrolled = 0, controlled = 1
Untreated hypertension	X5	Treated = 0, untreated = 1
Silent GERD	Y	Hypertension = 0, hypertension with silent GERD = 1

**Table 5:** Factors associated with the prevalence of silent GERD in patients with HT by multivariate logistic regression analysis.

Factors	Silent GERD *n* (%)	Controls *n* (%)	OR (95% CI)	*P* value
Education ≥ 12 years	8 (12.1)	71 (23.8)	0.22 (0.02–2.77)	0.205
Abdominal obesity	29 (43.9)	88 (29.5)	11.35 (4.92–26.18)	≤0.001^♦^
Prescription of calcium channel blockers	44 (66.7)	156 (52.3)	1.42 (0.70–2.92)	0.333
Controlled hypertension	9 (15.8)	78 (26.2)	0.02 (0.01–0.09)	≤0.001^♦^
Untreated hypertension	6 (9.1)	8 (2.7)	17.50 (3.65–83.87)	≤0.001^♦^

CI: confidence interval; OR: odds ratio; ^♦^*P* ≤ 0.001 compared with the control group, respectively.

## Abbreviations

BMI: Body mass index
EGD: Esophagogastroduodenoscopy
GERD: Gastroesophageal reflux disease
LA: Los Angeles
NERD: Nonerosive reflux disease
NYHA: New York Heart Association
RDQ: Reflux Disease Questionnaire.
